# Oviposition Preference of the Cabbage Root Fly towards Some Chinese Cabbage Cultivars: A Search for Future Trap Crop Candidates

**DOI:** 10.3390/insects11020127

**Published:** 2020-02-17

**Authors:** Fabrice Lamy, Laura Bellec, Amélie Rusu-Stievenard, Pauline Clin, Claire Ricono, Diane Olivier, Solène Mauger, Denis Poinsot, Vincent Faloya, Loïc Daniel, Anne Marie Cortesero

**Affiliations:** 1 Life science department, Campus de Beaulieu, Université de Rennes 1, UMR 1349 IGEPP, Avenue du Général Leclerc, CEDEX, 35042 Rennes, France; amelirusu@gmail.com (A.R.-S.); clin.pauline@gmail.com (P.C.); claire.ricono@etudiant.univ-rennes1.fr (C.R.); diane.olivier@hotmail.fr (D.O.); solene.mauger@univ-rennes1.fr (S.M.); denis.poinsot@univ-rennes1.fr (D.P.); anne-marie.cortesero@univ-rennes1.fr (A.M.C.); 2 INRA, UMR 1349 IGEPP, Domaine de la Motte, CEDEX, 35653 Le Rheu, France; laura.bellec35@gmail.com (L.B.); vincent.faloya@inra.fr (V.F.); loic.daniel@inra.fr (L.D.)

**Keywords:** Chinese cabbage, broccoli, *delia radicum*, *brassica rapa*, *brassica oleracea*, oviposition, host preference, multiple choice device

## Abstract

The development of integrated pest management strategies becomes more and more pressing in view of potential harmful effects of synthetic pesticides on the environment and human health. A promising alternative strategy against *Delia radicum* is the use of trap crops. Chinese cabbage (*Brassica* *rapa* subsp. *pekinensis* and subsp. *chinensis*) is a highly sensitive Brassicaceae species previously identified as a good candidate to attract the cabbage root fly away from other crops. Here, we carried out multi-choice experiments both in the laboratory and in field conditions to measure the oviposition susceptibilities of different subspecies and cultivars of Chinese cabbages as compared to a broccoli reference. We found large differences among subspecies and cultivars of the Chinese cabbage, which received three to eleven times more eggs than the broccoli reference in field conditions. In laboratory conditions, the *chinensis* subspecies did not receive more eggs than the broccoli reference. We conclude that *D. radicum* largely prefers to lay eggs on the *pekinensis* subspecies of Chinese cabbage compared to the *chinensis* subspecies or broccoli. Some *pekinensis* cultivars, which received over ten times more eggs than broccoli in the field, appear especially promising candidates to further develop trap crop strategies against the cabbage root fly.

## 1. Introduction

Phytophagous insects vary considerably both in their host range and selectivity within that host range and must face numerous choices in the different stages leading to host acceptance [1,2,3]. In many holometabolous insects like lepidopterans, coleopterans and dipterans, larval fitness is strongly dependent on adult females making the right choice when laying eggs. Thus, ovipositing females often exhibit a strong correlation between larval performance and plant host preference [4,5]. This observation supports the “mother-knows-best” hypothesis (or preference-performance hypothesis) which stipulates that, because of natural selection, females will acquire the ability to choose the best host available for their offspring [6]. However, the correlation between larval and female plant preference can be weak [7,8] or non-existent [9], leading females to ignore some suitable plants [10,11] or to lay eggs on unsuitable ones, resulting in poor larval development [12]. This “optimal bad motherhood” hypothesis [13] appears for example when the potentially most suitable plant host for larvae is unsuitable for adult fitness and thus unattractive to mothers. Oligophagy increases the likelihood that the same plant species is best for the fitness of adults and larvae, so that oligophagous insects are less likely to be “optimal bad mothers” than polyphagous ones [14,15].

Knowledge about insect host selection and preferences can be used to develop integrated pest management (IPM) strategies where the crop to be protected from a pest can be cultivated in combination with a far more attractive host plant used as a trap crop [16,17]. The trap crop diverts the pest from the main crop, which decreases economic damage. Moreover, if the trap crop is unsuitable for larvae (dead-end trap cropping), bad mother choice can be exploited to reduce the local population of the pest [18], limiting future crop damage. Numerous studies have revealed which plant hosts are preferred by major phytophagous insect pests and which are less appropriate for their larvae, with potential use in trap crop strategies [19,20,21]. However, preferences of insect pests have often been studied at the supraspecific or specific taxonomic levels while the wide infraspecific diversity created by plant breeding and varietal creation remains largely unexplored. This lack of information comes from the fact that conventional plant protection strategies based on pesticides do not require an extensive knowledge of plant susceptibility to insect pests and accordingly this trait is rarely considered in the plant breeding process. However, such knowledge is essential to develop new pest control strategies, such as trap cropping or push-pull, which rely on manipulating insect behaviour.

The cabbage root fly, *Delia radicum* L. (Diptera: Anthomyiidae) is an oligophagous insect of the Holarctic region which can successfully develop on various wild and cultivated species of Brassicaceae [22,23]. It can inflict severe crop damage in the absence of appropriate control measures [24,25]. Today, insecticides are still the main pest control solution but in most European countries, the number of registered products is decreasing due to their harmful impact on the environment and human health [26,27]. It has long been known that *D. radicum* prefers some cultivated hosts to others [28,29] with an especially strong preference for Chinese cabbage (*Brassica rapa* L.) [30]. This plant has therefore been proposed as a good trap crop candidate [31] and has more recently been used in two field studies aimed at developing a push-pull strategy to protect broccoli crops against this pest [32,33]. However, these studies only tested a single Chinese cabbage cultivar among many, so the relative efficiency of diverse Chinese cabbage cultivars as *D. radicum* trap crops remains to be explored. This approach is now necessary to determine the best trap crop candidate available against the cabbage root fly.

In this study, we compared the oviposition preference of the cabbage root fly among sixteen Chinese cabbage cultivars from the *pekinensis* and *chinensis* subspecies, with one broccoli cultivar as a reference. Cultivars were compared in multi-choice experiments in which all cultivars were present simultaneously, both in the laboratory (where the number of *D. radicum* females released was controlled) and in the field under natural infestation conditions.

## 2. Materials and Methods

### 2.1. Chinese Cabbage Diversity

Sixteen commercial cultivars of Chinese cabbage (*Brassica rapa* L.) were chosen among the wide diversity of the *pekinensis* and the *chinensis* subspecies and one cultivar of broccoli (*Brassica oleracea* L. var. *italica* cv. Marathon) was used as infestation reference (Table 1). This broccoli cultivar was commonly grown by farmers in Brittany and had been used in all our previous experimentations [33,34,35,36]. The selection of Chinese cabbages included some cultivars used in previous work carried out at our research unit (Kaboko, Tabaluga, Michihili and Joï Choi) and others for which data concerning *D. radicum* susceptibility were lacking.

### 2.2. Laboratory Choice Experiment

The culture of *D. radicum* used for the laboratory plant preference experiment was established in 2014 from a field population from Le Rheu, France, (48°6′31″ N, 1°47′1″ W). More than five thousand pupae were collected to start this colony and it was not refreshed. From 2014 until the experimentation in 2016, approximately 30 generations were maintained on rutabaga (*Brassica napus* subsp. *rapifera*) in a climatic room (16L:8D, 20 ± 2 °C and 60 ± 10% RH). After emergence, adults were placed in 30 cm or 50 cm cubic nylon cages and fed with water and a 1:1:1 mixture of milk powder, organic yeast and sugar. The females used were 7–10 days old and mated.

Plants were sown in individual pressed pot substrates (6 cm high × 4 cm Ø; i.e., 75 cm^3^) composed of white peat and perlite (Fertil SAS, Boulogne-Billancourt, France, ref: FERTISS 481.10). They were grown in plant culture chambers at 20–26 °C (DarkStreet DS90, Secret Jardin, Genval, Belgium) and lit by a metal halide bulb (Philips Master HPI-T Plus 400W/645, Philips, Amsterdam, The Netherlands). They were NPK-fertilized with a nutritive solution (Liquoplant Bleu^®^, Plantin SARL, Courthezon, France) diluted at 2‰ in water. Plants were used at the age of three weeks (2–3 true leaves stage).

Plant host preference in *D. radicum* was investigated in 50 cm cubic nylon cages (BugDorm, Taichung, Taiwan) using a multi choice set-up. Plants were used directly in their seeding pot placed in a Petri dish lid (5 cm Ø). The 17 cabbage cultivars (Table 1) were randomly arranged together in a circle (40 cm Ø; i.e., 7.4 cm between plants) and a water dispenser made from soaked cotton was positioned at its centre. Thirty-four females were released into the cage which was placed in a climatic chamber (Percival^®^ model E-41L2, Percival, Perry, IA, USA) (16L:8D, 20 ± 1 °C and 60 ± 10% RH). After 24 h, eggs were separated from plants and substrate by washing each plant and its whole individual substrate on top of a column of sieves (meshes: 2.5; 1; 0.25 mm). In order to count mature eggs remaining in the ovaries of the flies still alive at the end of the experiment and to estimate the total egg stock available, flies were dissected under a dissecting microscope. A total of 80 cages (1360 plants and 2720 flies) were run during this experiment (from 24 May 2016 to 05 August 2016). Only two cages were run simultaneously per day in the climatic chamber.

### 2.3. Field Choice Experiment

**Figure 1:**
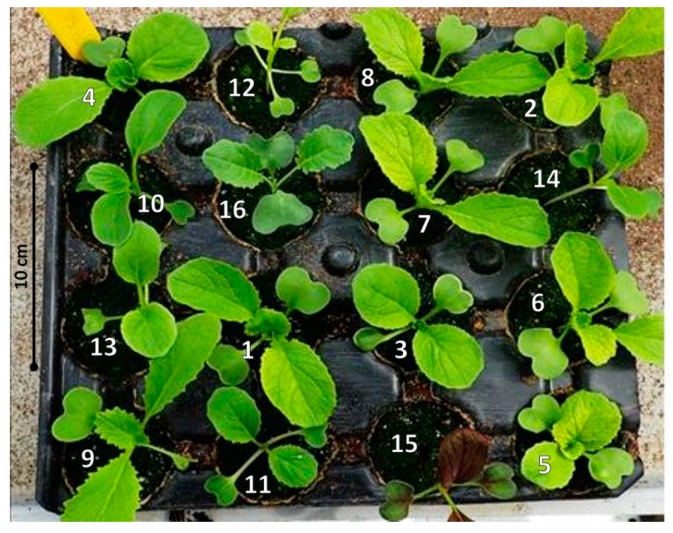
Photograph of a tray used for the plant preference experiment under natural infestation conditions. Numbers indicate cultivars (see Table 1). In this tray, plants 16, 7, 1 and 3 are central and all the others are peripheral.

In this experiment, 16 cabbage cultivars were used (Table 1). They were randomly seeded in 26 by 26 cm 16-cell trays (Soparco, Condé-sur-Huisne, France, ref: 5004). A tray always contained the 16 cultivars, so every tray used had the same plant composition, but a unique configuration. The 12 cells on the edge of the trays are considered peripheral and the 4 others, central (Figure 1). Each truncated cone-shaped cell (5.6 cm high, 4.8 cm top Ø, 3.8 cm bottom Ø; i.e., 82 cm^3^) was filled with substrate composed of white peat and sand (Premier Tech Horticulture, Vivy, France, ref: 992016F1). Plants were grown in a glasshouse at 18–24 °C. They were NPK-fertilized with Liquoplant Bleu^®^ at 2‰ as previously described. During 5 weeks, 30 trays were prepared each Monday and Thursday in order to be used three weeks later in the field (i.e., 2–3 true leaves stage).

The *D. radicum* plant preference experiment under natural infestation conditions took place in the experimental station “Terre d’Essais” at Pleumeur-Gautier (France: 48°48′19″ N, 3°9′4″ W). This station was chosen because it is located in a high *D. radicum* infestation area. The study was conducted during the first flight of *D. radicum* from 5 April to 10 May 2018. During this period, 28 to 30 trays aligned on a grid with one metre between them were placed on the bare ground in a 30 m wide field. The field was initially tilled and weeds were roughly kept under control manually (but not completely eradicated). No other brassicaceous crops were adjacent to this trial. After 72 or 96 h of exposure, the trays were removed and immediately replaced with new ones. The number of eggs laid on each plant was counted using the same process as described in the laboratory section. A total of 264 trays (4224 plants) were used during this experiment.

### 2.4. Statistical Analysis

All statistical analyses were performed with R software [37] version 3.6.2. In the laboratory experiments, the statistical unit (n) was the cage (n = 80) while it was the tray (n = 264) in the field; representing a randomized complete block design. For both the laboratory and field experiments, the effect of the cultivar on the number of eggs laid was tested using a Wald test applied on a generalized linear mixed model with a quasi-Poisson error distribution, a log link function (function “glmmPQL” of the package “MASS” version 7.3–51.5 [38]) and a type II analysis of variance (function “Anova” of the package “car” version 3.0–6 [39]). We included the date of experiment and the cage number as random factors in the model on laboratory data, whereas the date of sampling, the tray and the position of the plants in the tray were included as random factors in the model on field data. Then, pairwise comparisons of estimated marginal means (EMMs) were performed (function “emmeans” of the package “emmeans” version 1.4.3.01 [40]) using the Benjamini and Hochberg False Discovery Rate correction method for *p*-value adjustment [41]. The same procedure was followed to test the effect of the subspecies on the number of eggs laid. We also verified the effect of the seed coating (present vs. absent) and the plant position in the devices including the cultivar as random factor both on laboratory and field data. Position was “position on the circle” in the laboratory and “central vs. peripheral” in the field.

## 3. Results

### 3.1. Laboratory Choice Experiments

After 24 h of experiment, on average 89 ± 1% (mean ± S.E.) of the flies were still alive. More than a third of living flies (39 ± 2%) still had mature eggs in their ovaries, with an average of 24.2 ± 0.5 eggs per female. From the total number of mature eggs recovered, only 50 ± 2% were laid on the plants; the remaining eggs were present in the ovaries.

**Figure 2:**
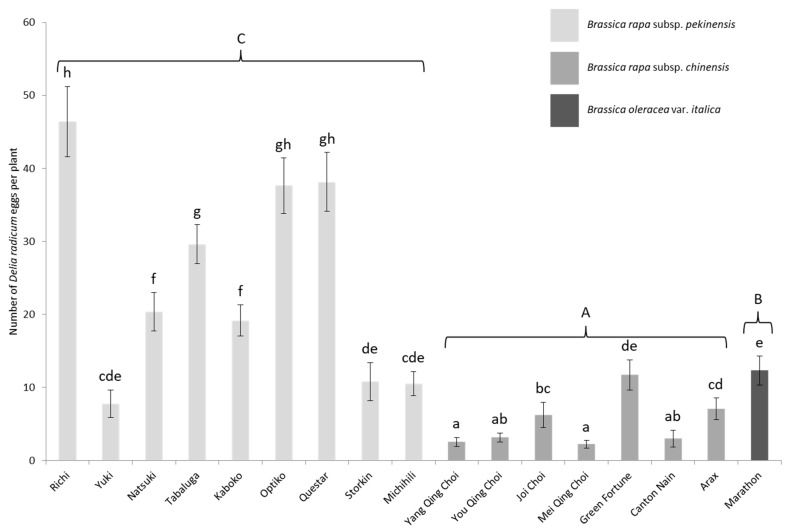
Mean number (±S.E.) of *D. radicum* eggs per plant depending on the cultivar in the laboratory experiment. Different capitalized letters indicate significant differences between subspecies (GLMM, FDR, *p* < 0.05) and lowercase letters, significant differences between cultivars (GLMM, FDR, *p* < 0.05).

On average five times more eggs were laid on *B. rapa* subsp. *pekinensis* than on *chinensis* and more than twice as many eggs were found on *B. oleracea* compared to *chinensis* (GLMM: χ^2^ = 230.08, df = 2, *p* < 0.001) (Figure 2). Neither the plant position inside the cage nor seed coating had a significant effect on oviposition (GLMM: χ^2^ = 25.38, df = 16, *p* = 0.06; χ^2^ = 0.03, df = 1, *p* = 0.87 respectively).

Chinese cabbage cultivars showed strikingly different oviposition susceptibility (GLMM: χ^2^ = 550.18, df = 16, *p* < 0.001) with some receiving more than ten times more eggs than others; *pekinensis* cv. Richi was the most infested cultivar (it was four times more infested than the *B. oleracea* reference) while *chinensis* cv. Mei Qing Choi and Yang Qing Choi were the least infested cultivars. Indeed, in this laboratory setting, *chinensis* cultivars scored poorly for fly oviposition since none received more eggs than the broccoli treatment and some even received significantly less (Figure 2).

### 3.2. Field Choice Experiment

**Figure 3:**
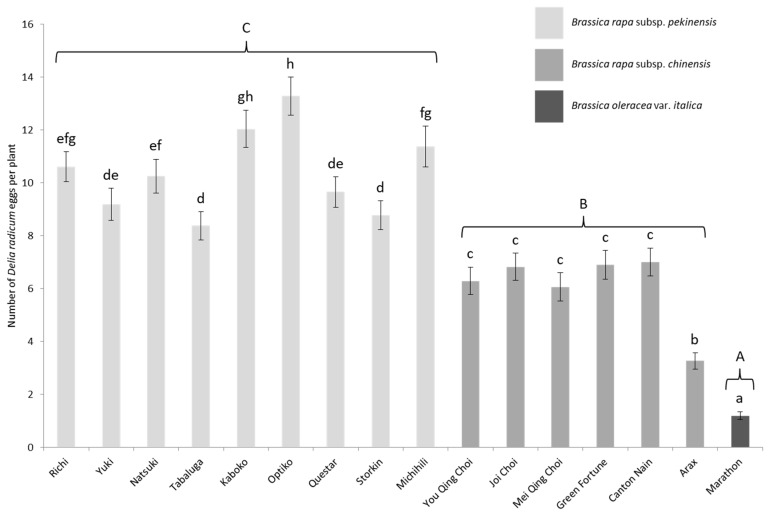
Mean number (±S.E.) of *D. radicum* eggs per plant depending on the cultivar in the field experiment. Different capitalized letters indicate significant differences between subspecies (GLMM, FDR, *p* < 0.05) and lowercase letters, significant differences between cultivars (GLMM, FDR, *p* < 0.05).

Significantly more eggs were laid on *B. rapa* subsp. *pekinensis* than on *chinensis* cultivars of the subspecies (Figure 3). *Pekinensis* received on average nine times more eggs than broccoli. Moreover, in sharp contrast to results from the laboratory experiment, *chinensis* also received far more eggs than the *B. oleracea* reference (Figure 3, GLMM: χ^2^ = 468.19, df = 2, *p* < 0.001). Seed coating had no significant effect on oviposition (GLMM: χ^2^ = 1.21, df = 1, *p* = 0.27) but plant position did: more eggs were laid on the peripheral plants of a tray than on the central ones (GLMM: χ^2^ = 18.79, df = 1, *p* < 0.001; mean ± S.E.: 8.5 ± 0.2 vs. 7.1 ± 0.3 respectively).

As in the laboratory, Chinese cabbage cultivars showed widely different oviposition susceptibilities in the field (GLMM: χ^2^ = 630.44, df = 15, *p* < 0.001). *B. rapa* subsp. *pekinensis* cv. Optiko was the most infested cultivar while the *B. oleracea* reference was the least infested. The number of eggs laid on broccoli was up to 11-fold lower than on Optiko. Although *pekinensis* cultivars still globally dominated *chinensis* ones in oviposition susceptibility, the contrast was nowhere near the same as in the laboratory situation, because in this field setting all *chinensis* cultivars received significantly more eggs than the *B. oleracea* reference (Figure 3).

## 4. Discussion

In both laboratory and field experiments, *D. radicum* females exhibited strong oviposition preferences for the *pekinensis* subspecies of Chinese cabbage, with some oviposition scores in the field being 10-fold higher than the broccoli reference. The oviposition susceptibility of the *chinensis* subspecies of Chinese cabbage appeared significantly lower than that of *pekinensis*, although this difference was stronger in the laboratory than in the field, where all *chinensis* cultivars tested received clearly more eggs than the broccoli reference. Our results confirm the general trend observed in some field studies regarding the high levels of attack of the *pekinensis* subspecies of the Chinese cabbage by *D. radicum* [28,30,42]. We also demonstrated and quantified the differences of *D. radicum* oviposition susceptibility at the cultivar level in Chinese cabbage

Some of the seeds we used were commercially film-coated with antifungal chemicals like thiram and iprodione in order to prevent damping-off. These chemicals reduce colonization of the seed by microorganisms and seed treatment might have been expected to reduce oviposition. For example, *D. radicum* lays fewer eggs on plants in which seeds have been disinfected with sodium hypochlorite [43]. However, no seed treatment effect was detectable in our laboratory or field experiments. Unpublished data referred to in [44] similarly suggest that root damage caused by *D. radicum* is similar whether seeds have been treated or not with Vitavax RS^®^ containing the fungicides thiram and carboxin.

In the laboratory experiment, we used a multiple-choice test rather than paired-tests to measure the relative oviposition preferences of the 17 cultivars tested because of the time and resources necessary to complete each of the 136 possible combinations. In closed experimental devices without host plant and odour, *D. radicum* females, which are at least eight days old, will deposit eggs even on neutral substrates like sand [45]. It is therefore not appropriate to compare oviposition susceptibility between cultivars in no-choice bioassays. In the field experiment, we chose to use a multiple-choice device as well. However, for practical reasons we could not use the same device in both experiments: in the laboratory, seventeen plants were placed in a circle while on the field only sixteen plants were placed on a square grid.

Host plant location and recognition by *D. radicum* females relies at long-range on volatile organic compounds (VOCs) [46,47] and at short-range on visual [48] and contact cues like glucosinolates (GSL) [49] or cabbage identification factors (CIFs) [50]. In the field experiment, *D. radicum* females had first to locate the trays of potted plants. The cabbage root fly approaches its host plant by flying upwind [51] and can detect hosts using volatile signals from at least 24 m [52]. In this field experiment, VOCs were probably the first cues used by females to locate and possibly discriminate host plants. In contrast, and because of the small size of our multi-choice laboratory device (only 0.5 m wide) when *D. radicum* females were released into it, they landed (i) on the base or on the walls of the cage, or (ii) directly on a plant. In this situation, they had direct visual or tactile contact with the plants from the beginning of the experiment so probably had less necessity to use volatile signals compared to the field experiment.

In the laboratory experiment, there was no effect of the plant position whereas for the field device, the four central plants had significantly fewer eggs than the peripheral ones. This result could be explained by a border effect. However, plant position did not mask fly responses to the different cultivars tested. This is because of (i) the large number of replicates, (ii) the randomization procedure in the assignation of the cultivar positions in the field (so all cultivars were placed in central and peripheral positions with similar frequencies) and (iii) the integration of the plant position as a random factor in the statistical model. The close proximity of cultivars allowed *D. radicum* females to move easily between them, facilitating choices. Also, in both our multi-choice devices, marked oviposition differences among cultivars revealed that our experimental setup allowed females to discriminate among them efficiently. In the field situation, the number of *D. radicum* females was not controlled and the number of eggs counted on each plant depended on several interacting parameters. First, plants that are more attractive (visually and because of their VOCs) will be visited more often by gravid females. Second, plants with more stimulating chemical contact profiles will more often trigger oviposition when visited. Third, in this species, contact with eggs of conspecifics also stimulates oviposition [53]. Accordingly, the plants chosen by the first female to visit a patch will further stimulate oviposition in the next females, further increasing the appeal of these plants, which partially explains the strongly aggregated distribution of eggs.

In both experiments, *pekinensis* cultivars received by far the most eggs. However, the hierarchy between *chinensis* and the *B. oleracea* reference was completely reversed between the laboratory and the field situation. In the laboratory, most *chinensis* cultivars received significantly fewer eggs than the broccoli reference while in the field they elicited far more oviposition events than the broccoli. This inverted hierarchy outlines once more that laboratory assays must only be considered as a handy, but sometimes fuzzy, predictor of field performance.

*Delia radicum* host discrimination is based on VOC profiles as well as visual characterization and phytochemical properties. These three parameters most probably explain the important oviposition differences we observed between subspecies and cultivars. Allyl isothiocyanate (AITC), hexyl acetate (HA) and dimethyl disulfide (DMDS) are VOCs know to affect the behaviour of gravid *D. radicum* females. AITC attracts females at low concentrations but becomes repellent at higher ones while HA is never attractive and also becomes repellent at high concentrations [54,55]. There is a higher concentration of AITC in the volatile profile of broccoli and *chinensis* than in *pekinensis*, while the concentration of HA is higher in the broccoli profile than in either *chinensis* or *pekinensis* [56]. DMDS reduces *D. radicum* oviposition when released at high concentration in the environment [57] and broccoli emits greater quantities of it compared to *pekinensis* or *chinensis* [56]. Together, these differences might explain the oviposition differences found between our three subspecies, however, data are still lacking regarding the variability of VOC profiles among the cultivars we used.

The shape, surface and colour of leaves can be discriminated by *D. radicum* females and influence landing and oviposition [45,58]. Among the cultivars of the Chinese cabbage subsp. *pekinensis*, during the plant production, we could only easily visually distinguish Michihili from the others as it had sharply serrated leaves (see Figure 1). However, this leaf shape did not seem to strongly affect *D. radicum* oviposition because Michihili received fewer eggs than the average of the other *pekinensis* cultivars in the laboratory while it received more eggs than the average of the other *pekinensis* cultivars in the field. Among *chinensis* cultivars, Arax was easily recognizable because of its red leaves and oviposition rates were low in both laboratory and field experiments. This result is in agreement with previous observations that *D. radicum* females lay fewer eggs on a red substrate compared to a bright green one [58]. Differences in plant architecture could also partially explain the oviposition differences between subspecies. *Pekinensis* cultivars were all stemless with a rosette architecture whereas *chinensis* cultivars and broccoli were both erect and caulescent.

After landing on a plant selected previously thanks to olfactory and visual cues, *D. radicum* females will proceed through a complex recognition pattern based on phytochemical characteristics [59]. Glucosinolates (GSL) and cabbage identification factors (CIFs) are surface compounds known to stimulate oviposition [49,60]. They are therefore, closely involved in host preference for oviposition [61]. Chemical analysis of four different parts (seeds, sprouts, shoots and roots) of broccoli (cv. Greendom), *chinensis* (cv. Chingen sai) and *pekinensis* (cv. Seoul) cultivars demonstrated that their GSL content was always lower in *pekinensis* [62]. GSL content also seems higher in *chinensis* than in *pekinensis* cultivars [63]. In *pekinensis*, the GSL content may however, vary widely among cultivars [64,65] and according to the age of the plant [66] with very distinctive profiles. Our results showed a significantly higher oviposition on *pekinensis*, which could suggest a preference of *D. radicum* females for plants with low GSL content. However, GSL content cannot be considered alone, since pure GSL is less stimulating than crude leaf extract [67] suggesting a synergistic effect between GSL and other compounds like CIFs. Unfortunately, we could not find any published study comparing CIF concentration among different Chinese cabbages or different species of Brassicaceae.

After one full day of laboratory experiment, 39% of the females still had mature eggs in their ovaries and only half of their global egg load had been laid on the plants. The arena used was small, so it seems unlikely that females failed to find a suitable plant in 24 h. However, the abundance of host cues could have made the selection between the seventeen cultivars complex so that females had to spend more time in choosing the most suitable host. Also, even the best hosts in the device might not have stimulated enough some gravid females with low oviposition pressure, in particular because the plants were small. This point is crucial and will have to be elucidated for the development of an efficient trap crop since such a plant must be capable of eliciting egg deposition in most females approaching the crop, including those that have only a moderate oviposition pressure.

Both our laboratory and field results confirm that in a choice situation, many more *D. radicum* eggs will be laid on Chinese cabbage subsp. *pekinensis* than on broccoli. Previous olfactory experiments demonstrated that *D. radicum* females exhibit strong preferences between hosts with different VOC profiles. In an earlier study, broccoli was less attractive than *pekinensis* (unspecified cultivar) which was one of the most attractive hosts tested [68]. Indeed, *pekinensis* (cv. Kaboko) is a more suitable host for development than broccoli (cv. Marathon), as measured using several life-history traits like egg to adult survival or adult size [33]. These results are the first to support the “mother-knows-best” hypothesis in *D. radicum*.

## 5. Conclusions

Our results confirm that the Chinese cabbage subsp. *pekinensis* might be a good candidate for further research and development in the perspective of a future integrated pest management strategy involving a trap crop to protect brassicaceous vegetables from *D. radicum*. In the modest diversity of cultivars that we tested, we observed strong differences in terms of oviposition preferences (that differed by ten-fold). Those differences are probably explained by chemical characteristics but further investigations are required to identify the key elements driving oviposition preference. Previously identified compounds such as AITC, GSL or CIFs are worth further investigations. Some Chinese cabbage cultivars such as Optiko and Kaboko (both of subsp. *pekinensis*) received over ten times more eggs than the broccoli reference in the field, making them potentially the best candidates among the 16 tested.

## Figures and Tables

**Table 1:** Varietal diversity of Chinese cabbages selected for plant laboratory and field choice experiments and broccoli used as infestation reference (F1 = F1 hybrid; Pop = population—i.e., mixture of genotypes); SV = Sakata Vegetable; GB = Graines Baumaux; LBG = La Bonne Graine. The asterisk indicates the variety excluded from the field experimentation.

Binomial Name	Subspecies/Variety	Cultivar (Genetics-Seed Provider)	Seed Coating	Number (Figure 1)
***Brassica rapa***	*pekinensis*	Richi (F1–SV)	Thiram	1
		Yuki (F1–SV)	Thiram	2
		Natsuki (F1–SV)	Thiram	3
		Tabaluga (F1–SV)	Thiram	4
		Kaboko (F1–GB)	None	5
		Optiko (F1–GB)	Iprodione	6
		Questar (F1–GB)	None	7
		Storkin (F1–GB)	None	8
		Michihili (Pop–LBG)	None	9
	*chinensis*	Yang Qing Choi (F1–SV) *	Thiram	–
		You Qing Choi (F1–SV)	Thiram	10
		Joi Choi(F1–SV)	Thiram	11
		Mei Qing Choi (F1–SV)	Thiram	12
		Green fortune (F1–GB)	Thiram	13
		Canton Nain (Pop–GB)	None	14
		Arax (Pop–GB)	None	15
***Brassica oleracea***	*italica*	Marathon (F1–SV)	None	16
